# Effective Removal of Acetaldehyde Using Piperazine/Nitric Acid Co-Impregnated Bead-Type Activated Carbon

**DOI:** 10.3390/membranes13060595

**Published:** 2023-06-12

**Authors:** Yu-Jin Kang, Yu-Jin Kim, Seong-Jin Yoon, Dong-Jin Seo, Hye-Ryeong Cho, Kyeongseok Oh, Seong-Ho Yoon, Joo-Il Park

**Affiliations:** 1 Department of Chemical & Biological Engineering, Hanbat National University, Daejeon 34158, Republic of Korea; yujin0581@naver.com (Y.-J.K.); yzzy0840@naver.com (Y.-J.K.); tjdwls7856@gmail.com (S.-J.Y.); tjehdwls0963@naver.com (D.-J.S.); ryeong2829@gmail.com (H.-R.C.); 2 Department of Chemical & Biological Engineering, Inha Technical College, Incheon 22212, Republic of Korea; kyeongseok.oh@inhatc.ac.kr; 3 Interdisciplinary Graduate School of Engineering Science, Kyushu University, Fukuoka 816-8580, Japan

**Keywords:** bead-type activated carbon, piperazine, nitric acid, surface modification, acetaldehyde removal

## Abstract

Acetaldehyde (CH_3_CHO) in the atmosphere is associated with adverse health effects. Among the various options for use in removing CH_3_CHO, adsorption is often employed because of its convenient application and economical processes, particularly when using activated carbon. In previous studies, the surface of activated carbon has been modified with amines to remove CH_3_CHO from the atmosphere via adsorption. However, these materials are toxic and can have harmful effects on humans when the modified activated carbon is used in air-purifier filters. Therefore, in this study, a customized bead-type activated carbon (BAC) with surface modification options via amination was evaluated for removing CH_3_CHO. Various amounts of non-toxic piperazine or piperazine/nitric acid were used in amination. Chemical and physical analyses of the surface-modified BAC samples were performed using Brunauer–Emmett–Teller measurements, elemental analyses, and Fourier transform infrared and X-ray photoelectron spectroscopy. The chemical structures on the surfaces of the modified BACs were analyzed in detail using X-ray absorption spectroscopy. The amine and carboxylic acid groups on the surfaces of the modified BACs are critical in CH_3_CHO adsorption. Notably, piperazine amination decreased the pore size and volume of the modified BAC, but piperazine/nitric acid impregnation maintained the pore size and volume of the modified BAC. In terms of CH_3_CHO adsorption, piperazine/nitric acid impregnation resulted in a superior performance, with greater chemical adsorption. The linkages between the amine and carboxylic acid groups may function differently in piperazine amination and piperazine/nitric acid treatment.

## 1. Introduction

Acetaldehyde (CH_3_CHO) is a volatile organic compound (VOC) in the atmosphere that is associated with adverse health effects. It is a factor in sick building syndrome, as it is found in wallpaper, furniture, cigarettes, and building materials [1,2,3,4,5,6,7,8]. Moreover, CH_3_CHO is odorous at a low concentration (0.09 mg/m^3^) and causes chest tightness, eye irritation, and respiratory tract irritation [9,10]. Therefore, indoor CH_3_CHO content should be regulated at <0.03 ppm, and an effective removal method for CH_3_CHO is required [11].

Several methods, such as absorption [12], condensation [13], biofiltration [14], and thermal oxidation [15], are commonly applied in removing VOCs such as CH_3_CHO, from gas streams. These methods are effective at relatively high VOC concentrations (>5000 ppmv) [16], but adsorption yields the optimal results in terms of energy cost, efficiency, and versatility with respect to the adsorbate [17]. Adsorption is one of the most efficient methods of removing VOCs, and various adsorbents are used, including carbon materials [18], silica [19], zeolites [20], and polymers [21]. Owing to their low costs and high stabilities, carbon materials are the most commonly used adsorbents in VOC removal [22]. Activated carbon, in particular, provides key advantages in adsorption because of its high porosity, large surface area, and rapid adsorption [23,24]. It is well-known that the intrinsic surface of activated carbon is hydrophobic. However, in many cases, the operation of activated carbon requires a hydrophilic environment such as water purification and wastewater treatment. Even VOC removal occurs in humid environments. Among the options of surface modification, acidic treatments are popular. Strong acids such as nitric acid are often used in surface modification. With the help of acidic treatment, the generation of carboxylic acid is noticeable along with hydroxyl groups. With the increasing need to remove specific chemicals such as aldehydes, the refined surface modification was necessitated with or without acidic treatment. Amination showed promising results in formaldehyde removal [18]. Previous studies have reported on the surface modification of activated carbon with amines that can be used to remove CH_3_CHO from the atmosphere. The amine-containing materials used in previous studies include aminobenzenesulfonic acids, ethylenediamine, diethylenetriamine, and amino acids [25,26,27]. However, these materials are toxic and can have harmful effects on humans when the modified activated carbon is used in air-purifier filters. Therefore, developing non-toxic amine-modified activated carbon is essential, but no related research regarding the adsorption of CH_3_CHO with non-toxic amines has been reported. In addition, the related references on acetaldehyde removal over activated carbon materials are summarized in Table 1.

**Figure 1:**
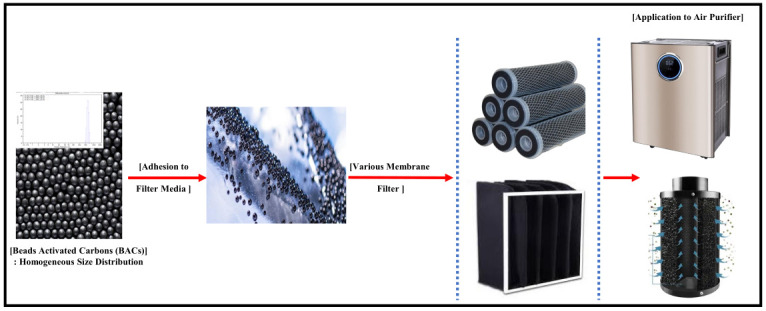
Conceptual illustration of BACs applied to air purification through systemizing membrane filtration.

In this study, we used a spherical bead-type activated carbon (BAC), which provides a pleasant working environment with no dust and high strength and fluidity. This type of BAC can be easily operated for adsorption and desorption, thereby making it feasible for recycling and helping for a circular economy. Specifically, compared to commercial granular activated carbon, BAC has a narrow-sized distribution. If BAC is applied to a BAC-incorporated membrane filter, a new design of the cartridge-type filter can be proposed. For a better understanding, the overall concept of BAC application is presented in Figure 1. Due to these properties, BAC is particularly useful as an air-purifier filter, and further research is necessary to explore its applicability [28]. The main aim of this study is to characterize the detailed adsorption behavior of CH_3_CHO over BAC modified with piperazine, which is a non-toxic amine. Moreover, the reaction mechanism of CH_3_CHO removal using the non-toxic amine-BAC is investigated in detail.

**Table 1:** The related research on acetaldehyde removal using activated carbon.

References	Activated Carbon Type	Impregnated Material	Mechanism
[29]	AC (Calgon, Norit, and Westvaco)	nitric acid	(1) When very small pores as close as the size of the acetaldehyde molecule and oxygen-containing groups are present (to a certain extent) within AC, the heat of adsorption reaches its maximum value. (2) A low density of surface groups can enhance the heat of adsorption, whereas extensive oxidation leads to a decrease in the strength of adsorption forces. This happens due to the blockage of the pore entrances containing functional groups and the decrease in the accessibility of hydrophobic surface where the dispersive interactions of hydrocarbon moiety can be enhanced.
[30]	AC (Calgon and Westvaco)	urea (450/950 °C)	(1) The adsorption forces are strong in small pores, and their volume governs the adsorbed amount. (2) The absorbed amount can be enhanced when functional groups bearing nitrogen are present. (3) These groups can provide additional adsorption centers when the small pores are filled with acetaldehyde molecules.
[26]	AC (coconut-shell and coal-base)	amine	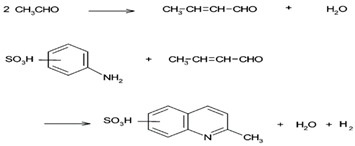
[31]	AC (corn grain)	KOH	(1) The effects of acetaldehyde adsorption on ACs were investigated in terms of textural properties, energetic heterogeneity, and surface chemistries. (2) The adsorption properties of water vapor were explained by the effect of the oxygen-containing groups on the surface of ACs over acetaldehyde adsorption. (3) The influences of pore size distribution (below 8 A˚) and energetic heterogeneity of ACs on acetaldehyde adsorption were highly predominant compared to that of specific surface area and surface chemistry.
[32]	AC (coconut base)	-	The study established a semi-quantitative relationship between pore size distribution and energy in relation to adsorption kinetics; the wider and more heterogeneous porosities resulted in higher rate constants for the resin-based carbon when compared to the ultramicroporous nutshell material.
[33]	ACF	metal oxide	ACF-K-20/5%MgO revealed three types of surface adsorption sites: one was assigned to physisorption on the surface O-containing carbon groups and two other sites are placed on a MgO surface and provide acetaldehyde chemisorption in two different modes.
[34]	ACF (cellulose base)	aniline-ethanol	(1) CH_3_CHO_(g)_ → CH_3_CHO_(AD)_ [Adsorption] (2) CH_3_CHO_(AD)_ + $\frac{1}{2}$O_2_ → CH_3_COOH [Oxidation] (3) 2CH_3_COOH → (CH_3_CO)_2_O + H_2_O [Dehydration] (4) (CH_3_CO)_2_O + C_6_H_5_NH_2_ → C_6_H_5_NCH_3_CO − CH_3_COOH
[35]	ACF (HDPE fiber)	Ag	(1) Ag particles were precipitated on the surface of ACF through interactive affinity, and the carbonyl group of AA is in creased to show that AA is adsorbed on the AC surface. (2) The AA adsorption of ACF and Ag/ACF composites performed in this study was suitable for the dose–response model, and the experimental data showing the asymmetric shape of the AA adsorption breakthrough curve for ACF and Ag/ACF composites were satisfactorily fitted.
[36]	AC and ACF	amine	(1) The high BET surface area provides more sites for acetaldehyde adsorption. (2) ACF has a systematic open macrostructure, which drives a low-pressure drop and allows fast adsorption without diffu sion hindrance.

## 2. Experimental Section

### 2.1. Materials and Sample Preparation

BAC (particle diameter of <0.1 mm) was obtained from ZEOBuilder (Seoul, Republic of Korea) and heated at 900 °C for 3 h under N_2_ flowing at 175 mL/min.

Piperazine, nitric acid, and ethyl alcohol were purchased from Samchun Pure Chemical (Pyeongtaek, Republic of Korea). Piperazine was prepared with ethyl alcohol, and nitric acid was prepared with distilled water. Aqueous stock solutions containing 1–10% (*w*/*v*) piperazine with or without 1% (*w*/*v*) nitric acid were prepared. The sample names differ according to the contents of the mixtures used (Table 2). The heated BAC was impregnated with the solution of piperazine with/without nitric acid, and the sample was then placed in a shaking water bath at 25 °C and shaken at 130 rpm for 24 h. After impregnation, the BAC was filtered and dried at 70 °C for 24 h. For comparison, piperazine was impregnated on coconut shell and coal-based activated carbons using the same procedure (Supplementary Information Figure S8).

### 2.2. Methods

#### 2.2.1. Preliminary Characterization of BACs

In order to investigate the characteristics of acetaldehyde removal for the BACs impregnated with piperazine/nitric acid, BARE-BAC was first examined by XRD, SEM, TEM, EDX, particle size distribution, as well as physical/chemical stability. All data is presented in the Supplementary Information (Figures S1–S7). In brief, BARE-BAC showed the amorphous phase (refer to XRD data in the Supplementary Information) without any crystallinity, therefore Raman characteristics were not necessary to verify its further crystallinity. BAC from a resin precursor contains a slight impurity of Si, characterized by SEM-EDX. Basically, approx. 1 wt% of impurity in activated carbon cannot affect its adsorption capacity. SEM morphology implies that the BACs were successfully synthesized from the resin precursor. The narrow window of particle size distribution in BARE-BAC ranged from 440 to 600 μm with a mean value of 512 μm, indicating homogeneous particle size compared with commercial granular activated carbon. It can be easily applied to the various type of membrane filter system. Moreover, the physical stability has been tested by attrition and abrasion (ASTM D4058-96 & SPENCE Method). Two tests were performed which showed the value of 99.77 with a deviation of 0.11. The acetaldehyde cycle test was carried out to confirm the chemical stability. The regeneration efficiency showed about 90% during 3 cycles of the adsorption–desorption process under the given operational conditions (200 ppm, GHSV: 37,500 h^−1^, 25 °C).

#### 2.2.2. N_2_ Sorption

N_2_ adsorption–desorption isotherms were obtained at 77 K using a surface area and pore size analyzer (BELSORP MAX G, Microtrac MRB, Osaka, Japan). Before the adsorption studies, the BAC was outgassed at 120 °C under vacuum for 8 h. The surface area was determined using the Brunauer–Emmett–Teller (BET) equation, the total pore volume was calculated at a relative pressure (P/P_0_) of 0.99, and the micropore volume was calculated using the Horvath–Kawazoe (HK) and t-plot methods.

#### 2.2.3. CHNS Elemental Analysis

A CHNS elemental analyzer (Flash 2000, Thermo Fisher Scientific, Waltham, MA, USA) with a thermal conductivity detector was used to examine the compositions of the BAC samples to determine the relative contents of C, H, N, and S as percentages.

#### 2.2.4. Fourier Transform Infrared (FTIR) Spectroscopy

FTIR spectroscopy was used to qualitatively evaluate the chemical structures of the BAC samples. The FTIR spectra were measured using an FTIR spectrometer (US/iS50, Thermo Fisher Scientific) in the frequency range of 400–4000 cm^–1^.

#### 2.2.5. X-ray Photoelectron Spectroscopy (XPS)

XPS was performed using an XP spectrometer (K-Alpha^+^, Thermo Fisher Scientific) with a monochromatic Al Kα (1486.6 eV) radiation source operated at 15 kW and 50 W. Prior to the analysis, the samples were outgassed at room temperature until the system pressure reached 5.2 × 10^–9^ torr. High-resolution spectra were collected at a constant pass energy of 29.35 eV over an area with a diameter of 4000 μm. The amount of each element (C, O, N, and S) was calculated using individual spectrum, and the energy scale was calibrated using the C 1s photoelectron line at 285.0 eV.

#### 2.2.6. CH_3_CHO Adsorption

**Figure 2:**
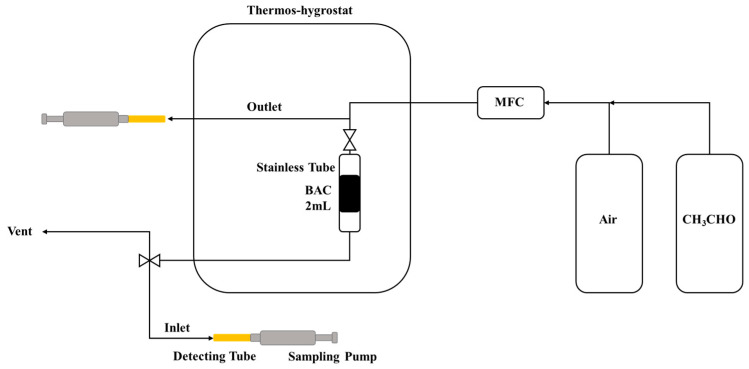
Schematic illustration of our CH_3_CHO removal apparatus for lab-scale tests.

Figure 2 schematically illustrates the continuous flow system employed in evaluating CH_3_CHO removal using the BAC samples. Tests were conducted at 10 °C using a thermo-hygrostat (climatic chamber, Weiss Technik, Rieskirchen-Lindenstruth, Germany), and the BAC sample (2 mL) was placed in a stainless steel column. The CH_3_CHO gas was 1000 ppm CH_3_CHO in N_2_, which was then mixed with air to yield a CH_3_CHO concentration of 200 ppm. Gas analysis was performed using a sampling pump (GV-100S, Gastec, Ayase, Japan) and tube (92/92M/92L, Gastec). The breakthrough adsorption capacity of CH_3_CHO (W_ad_ [mg/g]) was calculated by integrating the area under the breakthrough curve using the flow rate, CH_3_CHO concentration, time, and adsorbent mass. All adsorption tests were stopped at C_out_/C_in_ = 1 (C_out_ [ppm]: outlet concentration of CH_3_CHO; C_in_ [ppm]: inlet concentration of CH_3_CHO).

#### 2.2.7. Thermal Regeneration

The modified BACs were thermally regenerated in a muffle furnace (Daihan Scientific, Wonju, Republic of Korea). After the CH_3_CHO adsorption studies, the spent BAC samples (approximately 2 mL) were heated from room temperature to 453 K (heating rate: 5 K/min) and maintained at this temperature for 2 h in air.

## 3. Results

### 3.1. Characterizations of BAC

#### 3.1.1. Textural Structure

The textural properties of the BAC samples, which were determined using the N_2_ adsorption–desorption isotherms, are summarized in Table 3. The BET surface areas (S_BET_) decrease in the following order: BARE-BAC > piperazine/nitric acid-co-impregnated BACs > piperazine-modified BACs. Piperazine treatment decreases the S_BET_ of BARE-BAC, but the co-impregnation with piperazine and nitric acid minimizes the decrease, likely because the nitric acid molecules adsorbed on the pore surfaces continue to penetrate the pore walls during impregnation. However, as the piperazine content increases, the pores of the BAC are blocked, and S_BET_ decreases. BARE-BAC displays the highest S_BET_ (1442.1 m^2^/g) and the highest micropore and total pore volumes (0.6189 and 0.6284 cm^3^/g, respectively), which are almost 6–77% higher than those of the modified BACs. Therefore, chemical factors have a greater effect on CH_3_CHO adsorption than physical factors.

The piperazine/nitric acid-co-impregnated BACs exhibit larger micropore volumes than the piperazine-modified BACs. Overall, co-impregnation with piperazine and nitric acid should result in different modifications of the BAC surface compared to those caused by piperazine modification of the BAC.

#### 3.1.2. CHNS Elemental Analysis

The contents of C, H, N, and S in the samples were measured using a CHNS elemental analyzer, and the results are shown in Table 4. The N and H contents increase after treatment with piperazine and nitric acid, respectively, and the introduced amine (–NH) groups are critical in CH_3_CHO removal via chemisorption [26,27,34].

#### 3.1.3. Chemical Characterization

**Figure 3:**
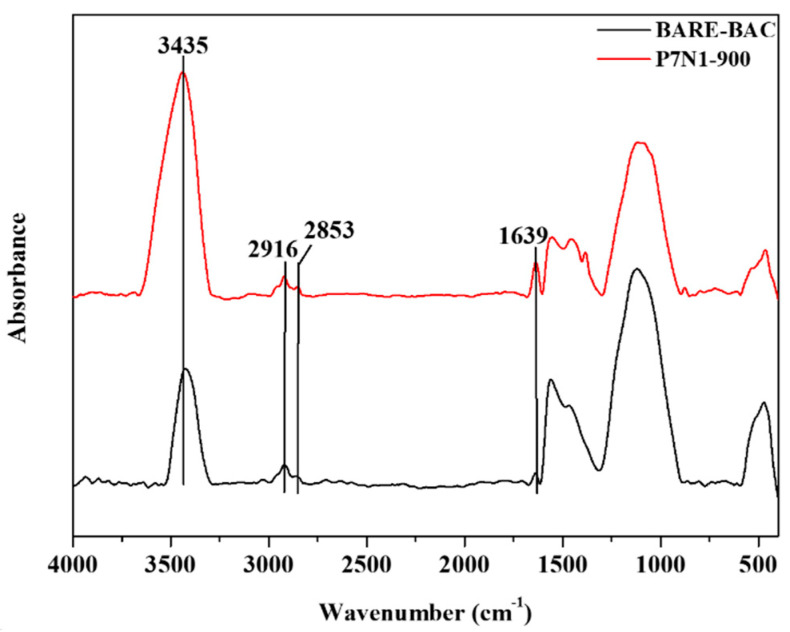
FTIR spectrum of BARE-BAC and P7N1-900.

Figure 3 shows the FTIR spectra of BARE-BAC and P7N1-900, which adsorbs the highest amount of CH_3_CHO. The characteristic peaks attributed to the functional groups are almost identical in the two spectra. The O–H and N–H bonds result in noticeable peaks, and Table 5 summarizes the intensity data of the functional groups. The spectrum of P7N1-900 displays peaks with stronger intensities the O–H and amine functional groups representing treatment with piperazine and nitric acid. Penchah et al. [37] reported that nitric acid increases the carboxylic acid (COOH) content on the surface of activated carbon, and piperazine is linked to COOH. Therefore, the number of OH groups of P7N1-900 is increased approximately 2-fold on account of using nitric acid, and the number of amine groups is increased approximately 4.2-fold on account of using piperazine. In addition, no change in the structure of BAC is observed, as there is almost no change in the –CH_2_ groups.

Ryu et al. [34] reported that the amine functional groups on activated carbon lead to a higher CH_3_CHO adsorption efficiency, and thus, the amine groups should increase CH_3_CHO adsorption.

#### 3.1.4. XPS

**Figure 4:**
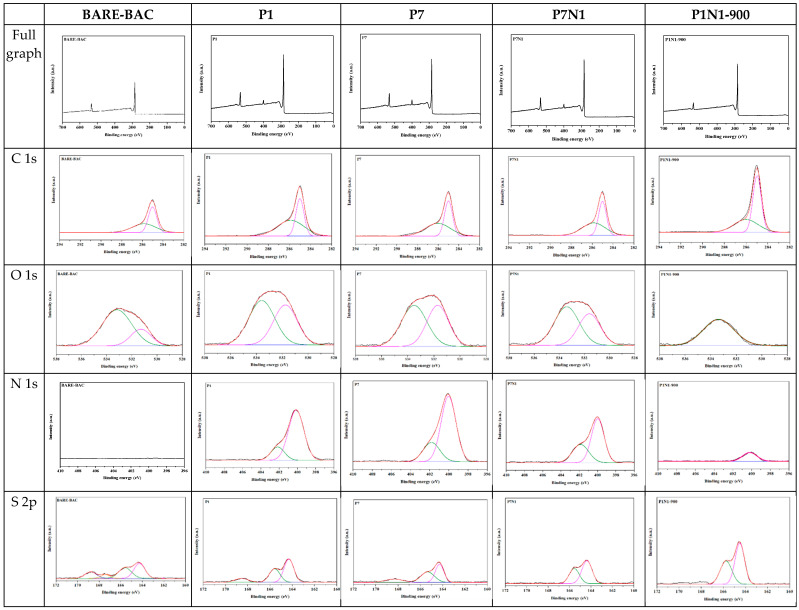

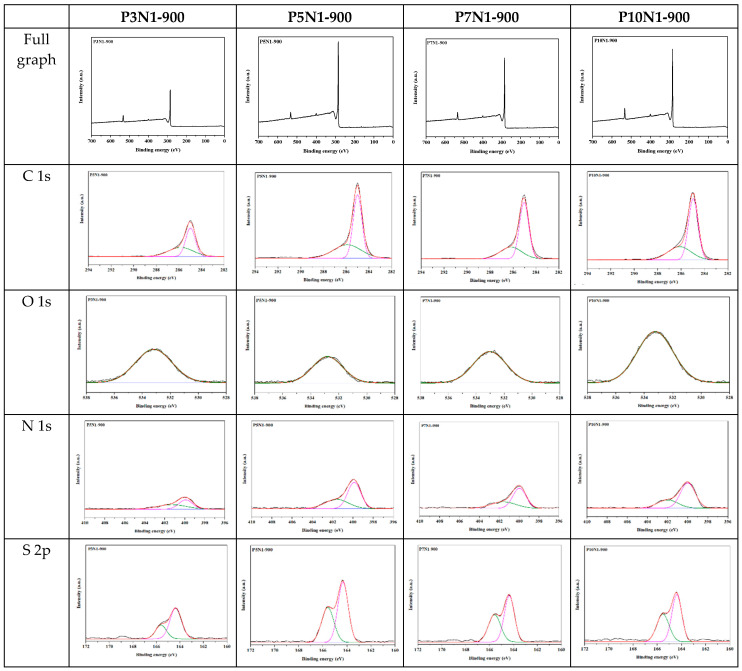
High resolution XPS spectra of C 1s, O 1s, N 1s, and S 2p for all BAC samples. (black line: experimental value, red line: fitting value).

XPS was used to investigate the chemical states of the elements in the BAC samples. The deconvoluted spectra are shown in Figure 4, and the relative abundances of C, O, N, and S are presented in Table 6.

After modification with piperazine, the number of N bonds increases, whereas the number of bonds involving S decreases. After heating and modification with piperazine and nitric acid, the number of N bonds increases, and the number of bonds involving O and S decreases.

The various N components in BAC were further determined by fitting the N 1s spectra. As shown in Figure 4 and Table 5, deconvolution of the N 1s spectrum reveals the presence of N–(C)_3_ (tertiary nitrogen, secondary amine) and C–N^+^O–C (oxidized nitrogen functionalities). After piperazine treatment, the number of assigned N bonds increases, particularly the N–(C)_3_ content, and thus, when only piperazine is used, it coats the BAC surface. By contrast, in the BAC treated with piperazine and nitric acid, the content of C–N^+^O–C increases by >50% and then decreases as the piperazine content increases. Thus, COOH groups are formed on the surface of the BAC via the addition of nitric acid, and piperazine is linked to these groups. The piperazine content of P1N1-900 is excessively low to link to the COOH groups, and thus, the content of N–(C)_3_ is 100%. Conversely, in P3N1-900, piperazine and the COOH groups are linked, increasing the content of C–N^+^O–C. In P5N1-900, the COOH groups and piperazine are linked, the remaining piperazine is coated on the surface of the BAC, and the content of N–(C)_3_ is increased. N atoms doped onto the BAC surface may react with CH_3_CHO to enhance its adsorption [30].

As shown in Figure 4 and Table 5, deconvolution of the O 1s and S 2p spectra reveal the presence of O–C/O–S (in phenol/epoxy or thioether/sulfonic acid), O=C/O=S (in carboxy/carbonyl or sulfoxides/sulfones), C–S–C (in sulfides), R–S–S–OR (in thioethers), R_2_–S=O (in sulfoxides), and R–SO_2_–R (in sulfones). During heating, the high temperature may convert O=C/O=S to O–C/O–S, and R_2_–S=O and R–SO_2_–R in P1N1-900–P10N1-900 are converted to C–S–C via heating. S doped on the BAC surface may also react with aldehydes to increase the HCHO adsorption performance [39].

**Table 6:** Results of the deconvolution of the XPS spectra of C 1s, O 1s, N 1s, and S 2p (in bold—atomic % of specific elements).

Bond Assignment	Energy [eV]	BARE-BAC [%]	P1 [%]	P7 [%]	P7N1 [%]	P1N1-900 [%]	P3N1-900 [%]	P5N1-900 [%]	P7N1-900 [%]	P10N1-900 [%]
**C 1s**										
C-C sp^2^	284.8	51.15	41.23	46.46	47.57	62.49	55.21	60.32	69.02	66.84
C-O (phenol, alcohol, ether), C=N (amine, amide)	286.0–286.3	48.85	58.77	53.54	52.43	37.51	44.79	39.68	30.98	33.16
**O 1s**										
O-C/O-S (in phenol/ epoxy or thioethers/sulfonic)	533.3–533.6	77.78	55.29	54.16	59.19	100	100	100	100	100
O=C/O=S (in carboxy/carbonyl or sulfoxides/sulfones)	532.0–532.5	22.22	44.71	45.84	40.81	-	-	-	-	-
**N 1s**										
N-(C)_3_ (tertiary nitrogen, secondary amine)	399.1–400.0	-	83.51	76.46	68.18	100	100	63.78	67.99	70.61
C-N^+^O-C (oxidized nitrogen functionalities)	402.3	-	16.49	23.54	31.82	-	-	36.22	32.01	29.39
**S 2p**										
C-S-C (in sulfides); R-S-S-OR (in thioethers)	164.5–166.0	81.74	96.35	93.56	93.56	100	100	100	100	100
R_2_-S=O (in sulfoxides)	167.0–167.3	2.28	-	-	-	-	-	-	-	-
R-SO_2_-R (in sulfones)	168.4–168.6	15.98	3.65	6.44	6.44	-	-	-	-	-

Sources: Adapted from [40].

### 3.2. CH_3_CHO Adsorption

**Figure 5:**
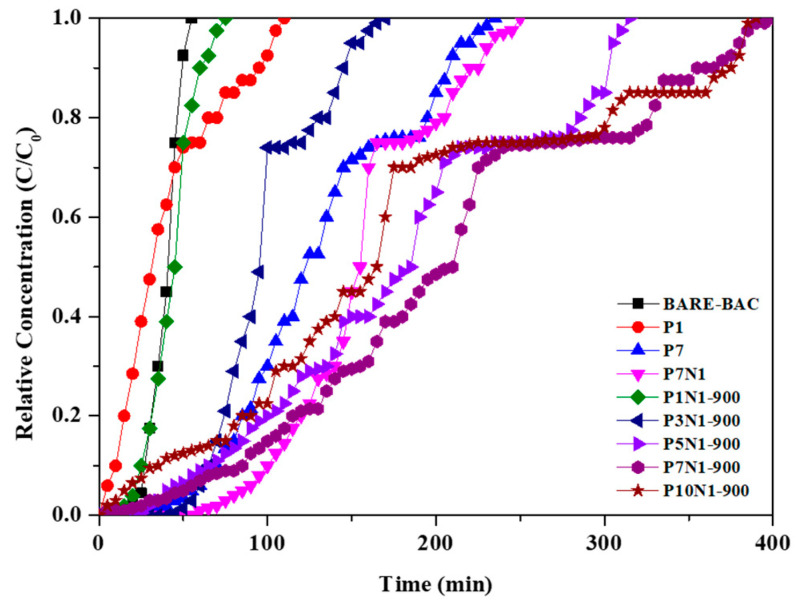
CH_3_CHO breakthrough curves of all BAC samples.

**Figure 6:**
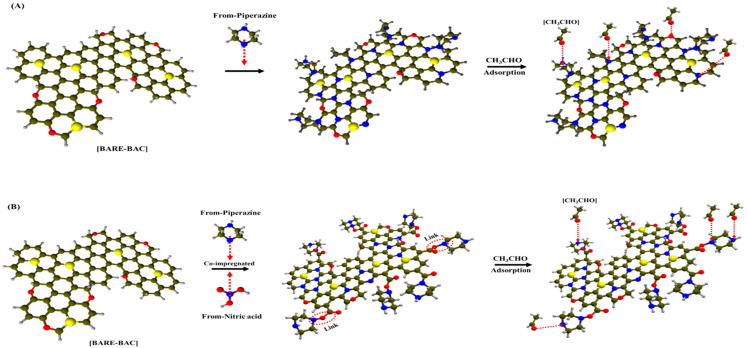
Conceptual diagram of (**A**) piperazine impregnated and (**B**) piperazine and nitric acid co-impregnated on the surface of BAC.

Figure 5 shows the CH_3_CHO breakthrough curves measured at 10 °C in dry conditions, and the calculated adsorption parameters are listed in Table 6. The modified BAC samples display higher W_ad_ values, as shown in Table 7, than that of the unmodified BAC: 17.17, 18.73, 50.05, 57.44, 18.84, 54.48, 62.95, 72.34, and 61.38 mg/g for BARE-BAC, P1, P7, P7N1, P1N1-900, P3N1-900, P5N1-900, P7N1-900, and P10N1-900, respectively. The samples co-impregnated with piperazine and nitric acid generally exhibit higher W_ad_ values than those of the samples treated only with piperazine (P1 and P7). P7N1-900, in particular, adsorbs the largest amount of CH_3_CHO, which is 4.2-fold higher than that adsorbed by BARE-BAC. Physical factors, such as the high S_BET_ values of the modified BAC samples and abundant micropores, likely contribute to the adsorption performances of the co-impregnated BAC samples. Moreover, heating destroys the functional groups on the surface of the BAC and ensures that the active sites comprising piperazine and nitric acid are evenly distributed. As the piperazine content increases, the amount of adsorbed CH_3_CHO increases, but when the piperazine content is excessive, as in P10N1-900, the pores of the BAC are blocked and the adsorption performance is reduced. Chemical factors, such as the addition of nitric acid, result in higher CH_3_CHO adsorption. The chemical reactions between CH_3_CHO and linked piperazine explain the increased CH_3_CHO adsorption levels of the BAC samples co-impregnated with piperazine and nitric acid compared to those of the other samples. This is corroborated by the XPS spectra, which show that CH_3_CHO reacts strongly with C–N^+^O–C (oxidized nitrogen functionalities) and N–(C)_3_ (tertiary nitrogen, secondary amine). The reaction with CH_3_CHO is stronger when both types of bonds are present compared to that when one type is present. Figure 6 shows the comprehensive mechanisms of amine modification of the BAC and CH_3_CHO adsorption on the modified BAC surface.

### 3.3. Effect of Thermal Regeneration on CH_3_CHO Adsorption

**Figure 7:**
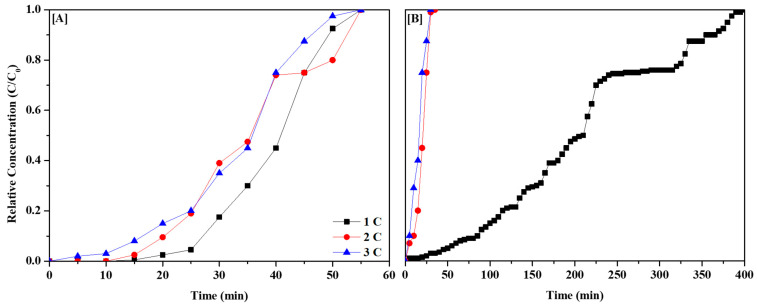
CH_3_CHO regeneration efficiency of 3 cycles: BARE-BAC (**A**) and P7N1-900 (**B**).

Finally, we evaluated the thermal regeneration efficiencies of BARE-BAC and P7N1-900 after CH_3_CHO adsorption. The BAC samples were subjected to three cycles of CH_3_CHO adsorption and regeneration. Thermal regeneration was performed by heating the samples at 5 K/min to 453 K in a muffle furnace and maintaining this temperature for 2 h in air. Figure 7 shows the breakthrough curves obtained under dry conditions, and Table 8 presents the data. The results of Cycles 2 and 3 exhibit similar trends to those shown in Figure 5. As shown in Table 8, the respective regeneration efficiencies of BARE-BAC and P7N1-900 are 86.78% and 8.77%. Amine modification significantly reduces the regeneration efficiency of BARE-BAC by a factor of 10. BARE-BAC exhibits a high ratio of physical adsorption, whereas P7N1-900 displays a high ratio of chemical adsorption. BARE-BAC physisorbs CH_3_CHO and desorbs it well in all cycles. By contrast, P7N1-900 chemisorbs CH_3_CHO in Cycle 1 but does not desorb well, and thus, the CH_3_CHO adsorption declines significantly in Cycle 2. Meanwhile, the amounts of CH_3_CHO adsorbed in Cycles 2 and 3 are similar.

## 4. Conclusions

Customized BAC was heated at 900 °C and impregnated with piperazine or binary piperazine/nitric acid to enhance CH_3_CHO removal via adsorption. The amount of piperazine was varied from 1 to 10% (*w*/*v*) and nitric acid was used at 1% (*w*/*v*). When only piperazine was used, the pore and micropore volumes of the modified BACs decreased with increasing piperazine content. The S_BET_ of BARE-BAC was 1442.1 m^2^/g, and that of P7 was reduced to 794.5 m^2^/g (modified BAC with 7 % *w*/*v* impregnation of piperazine). Meanwhile, co-impregnation resulted in a minimized decrease in S_BET_, e.g., 1347.2 m^2^/g for P1N1-900 (1% *w/v* each of piperazine and nitric acid, followed by heating at 900 °C). This suggests that nitric acid penetrates into the BAC microstructure to form pores of larger size and volume. However, the qualitative chemical structures of BARE-BAC and P7N1-900 (impregnation with 7% *w/v* piperazine and 1% *w/v* nitric acid, followed by heating at 900 °C) were not significantly different according to FTIR spectroscopy. Elemental analysis revealed that amine treatment increased the N contents of the BAC samples. When CH_3_CHO adsorption was performed at 10 °C under dry conditions, the adsorption performance of P7N1-900 increased 4.2-fold compared to that of BARE-BAC. This suggests that the chemical factors of BAC have a greater effect on CH_3_CHO adsorption than the physical factors. In regard to the impregnation with piperazine only (P1 and P7), the lower CH_3_CHO adsorption capacities could be explained by linkages to hidden amines and intrinsic COOH groups, resulting in negative effects on adsorption. By contrast, P1N1-900 and P7N1-900 should exhibit linked piperazine-amines but still contain carboxylic acids on the surfaces of the BAC samples, resulting in increased levels of CH_3_CHO adsorption. The CH_3_CHO adsorption capacity of BARE-BAC was 17.17 mg/g, and those of P7 and P7N1-900 increased to 50.05 and 72.34 mg/g, respectively. The XP spectra indicated that N–(C)_3_ (tertiary nitrogen, secondary amine) and C–N^+^O–C (oxidized nitrogen functionalities) were present throughout the amine-treated BAC samples. The presence of C–N^+^O–C indirectly indicated that the amines were linked to COOH groups, which were mostly generated via nitric acid treatment. An increased amine content resulted in higher concentrations of N–(C)_3_, and thus, amine treatment introduced primary and secondary NH functional groups into the modified BAC and facilitated CH_3_CHO adsorption. Although CH_3_CHO adsorption was only evaluated under flow conditions, the modified BAC samples should display high CH_3_CHO adsorption capacities even under static conditions.

## Figures and Tables

**Table 2:** Description of each sample.

Sample	Piperazine % [*w*/*v*%]	Nitric Acid % [*w*/*v*%]	Heat Treatment Temp. [°C]
P1	1	-	-
P7	7	-	-
P7N1	7	1	-
P1N1-900	1	1	900
P3N1-900	3	1	900
P5N1-900	5	1	900
P7N1-900	7	1	900
P10N1-900	10	1	900

**Table 3:** Textural properties of BAC samples.

Sample	S_BET_ [m^2^/g]	S_Micro_ [m^2^/g]	V_Total_ [cm^3^/g]	V_Micro_ [cm^3^/g]	Average Pore Diameter [nm]
BARE-BAC	1442.1	1437.3	0.6284	0.6189	1.7429
P1	921.5	916.9	0.4123	0.4033	1.7898
P7	794.5	791.0	0.3533	0.3462	1.7788
P7N1	1141.3	1137.1	0.5001	0.4916	1.7528
P1N1-900	1347.2	1341.2	0.5905	0.5788	1.7533
P3N1-900	1275.3	1270.2	0.5612	0.5508	1.7602
P5N1-900	1191.6	1185.8	0.5259	0.5142	1.7652
P7N1-900	1115.3	1110.0	0.4818	0.4711	1.7281
P10N1-900	983.8	979.3	0.4390	0.4298	1.7850

**Table 4:** Contents of C, H, N, and S in BAC samples.

Sample	C [%]	H [%]	N [%]	S [%]
BARE-BAC	93.84	0.45	* ND	1.31
P1	80.18	2.16	0.63	1.01
P7	87.00	1.64	3.52	1.13
P7N1	85.90	1.74	1.89	1.21
P1N1-900	83.02	2.30	0.39	0.97
P3N1-900	93.39	0.79	1.23	1.25
P5N1-900	81.99	2.67	1.52	0.94
P7N1-900	83.21	2.21	2.13	0.98
P10N1-900	80.89	2.77	2.67	0.89

* ND: No data.

**Table 5:** Intensity of BARE-BAC and P7N1-900′s functional groups.

Band Position [cm^−1^]	Component	Intensity	
		BARE-BAC	P7N1-900
3435	O-H	3.40	6.95
2916	Saturated aliphatic CH_2_	0.42	0.46
2853	Saturated aliphatic CH_2_	0.16	0.21
1639	Amie, primary/secondary NH	0.29	1.23

Sources: Adapted from [38].

**Table 7:** CH_3_CHO adsorption parameters on all BAC samples.

Sample	C_in_ [ppm]	W_ad_ [mg/g]
BARE-BAC	200	17.17
P1	200	18.73
P7	200	50.05
P7N1	200	57.44
P1N1-900	200	18.84
P3N1-900	200	54.48
P5N1-900	200	62.95
P7N1-900	200	72.34
P10N1-900	200	61.38

**Table 8:** CH_3_CHO regeneration efficiency of three cycles.

Sample	Number of Cycle	Adsorption Amount [mg/g]	Regeneration Efficiency of 3 Cycles [%]
BARE-BAC	1	17.17	86.78
	2	15.89	
	3	14.90	
P7N1-900	1	72.34	8.77
	2	6.13	
	3	5.97	

## Data Availability

The data presented in this study are available upon request from the corresponding author.

## Supplementary Materials

The following supporting information can be downloaded at: https://www.mdpi.com/article/10.3390/membranes13060595/s1, Figure S1: SEM Morphology of Bare BAC, Figure S2: TEM Morphology of Bare BAC (After Grinding/Crushing BAC), Figure S3: XRD Patterns of Bare BAC, Figure S4: SEM-EDX of Bare BAC, Figure S5: Attrition and Abrasion Test of Bare BAC (Physical Stability), Figure S6: Acetaldehyde Cycle Test over Bare BAC, Figure S7: Particle Size Distribution of Bare BAC, Figure S8: CH_2_CHO Adsorption Parameters on Cucunut Shell and Coal Based Activated Carbon Samples.
