# Supplementary material for: Effective Removal of Acetaldehyde Using Piperazine/Nitric Acid Co-Impregnated Bead-Type Activated Carbon
Source: Membranes (Basel). 2023 Jun 12;13(6):595. doi: 10.3390/membranes13060595 (PMC10302408; doi:10.3390/membranes13060595)
Supplement: Supplementary file 1 [file membranes-13-00595-s001.zip › membranes-2417201-supplementary.pdf]

Supplementary Information

Supplementary Information-1: SEM Morphology of Bare BAC

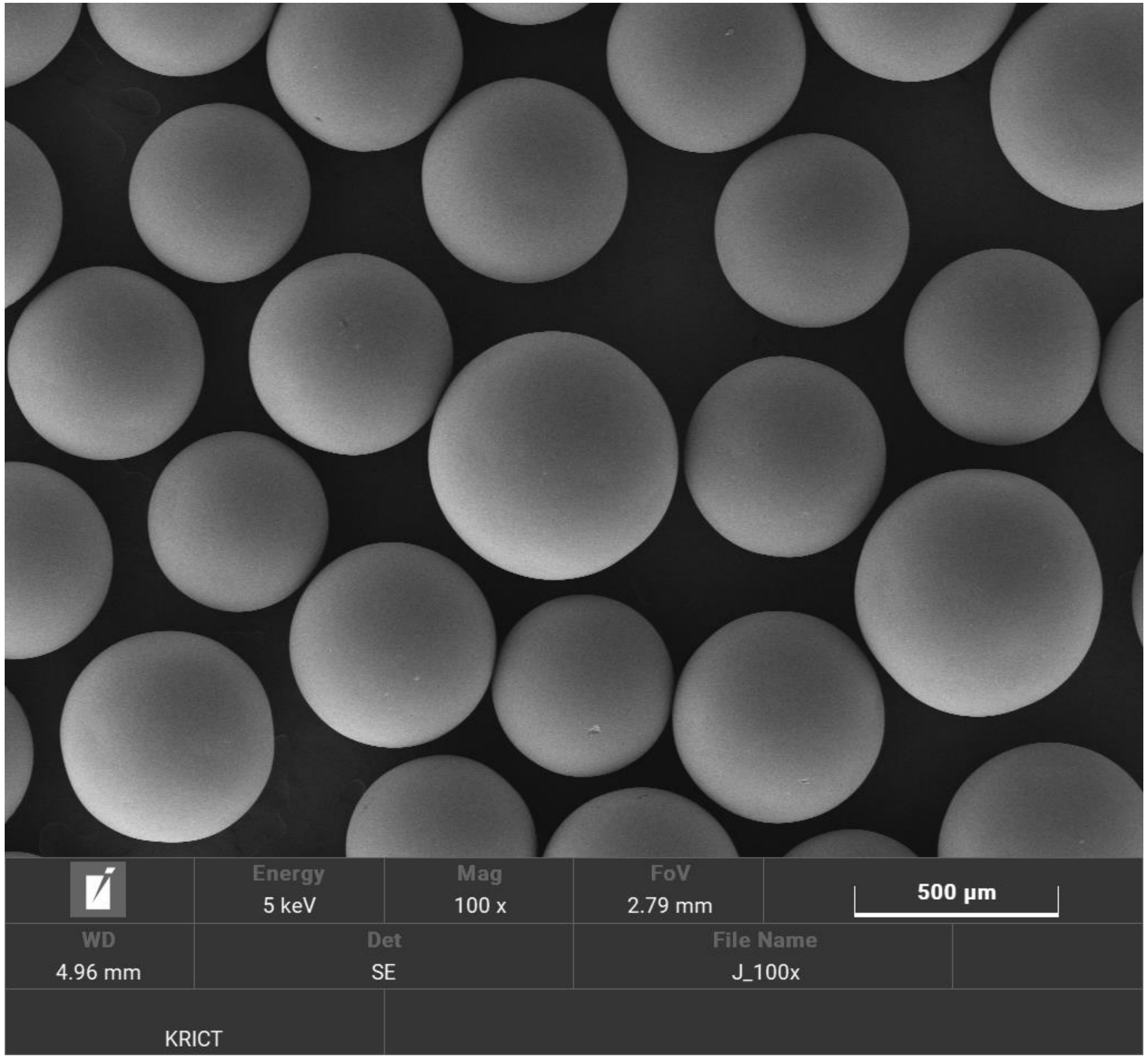

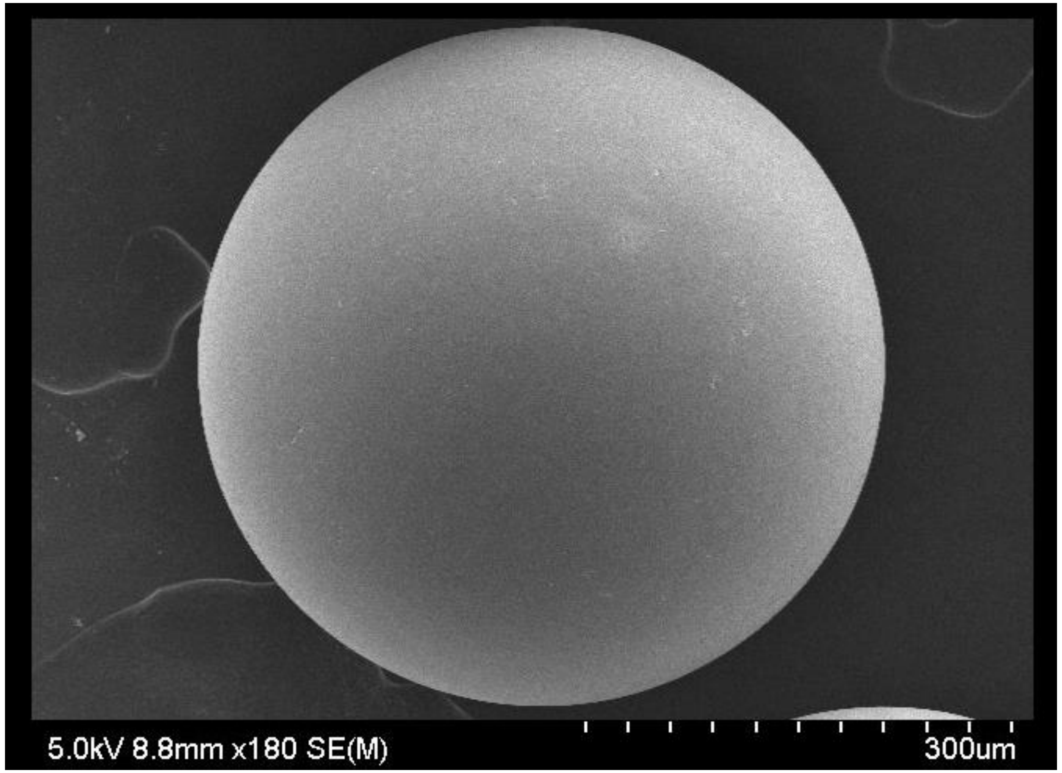

Supplementary Information-2: TEM Morphology of Bare BAC (After Grinding/Crushing BAC)

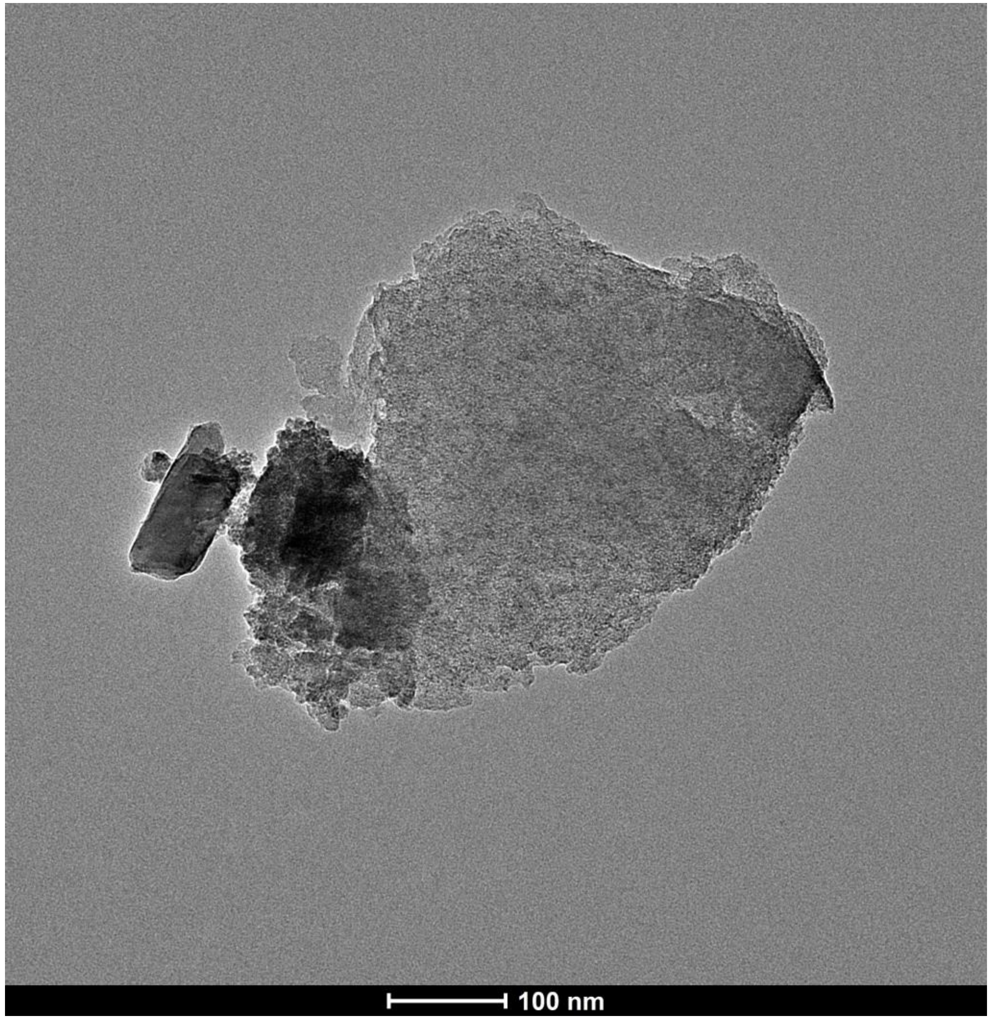

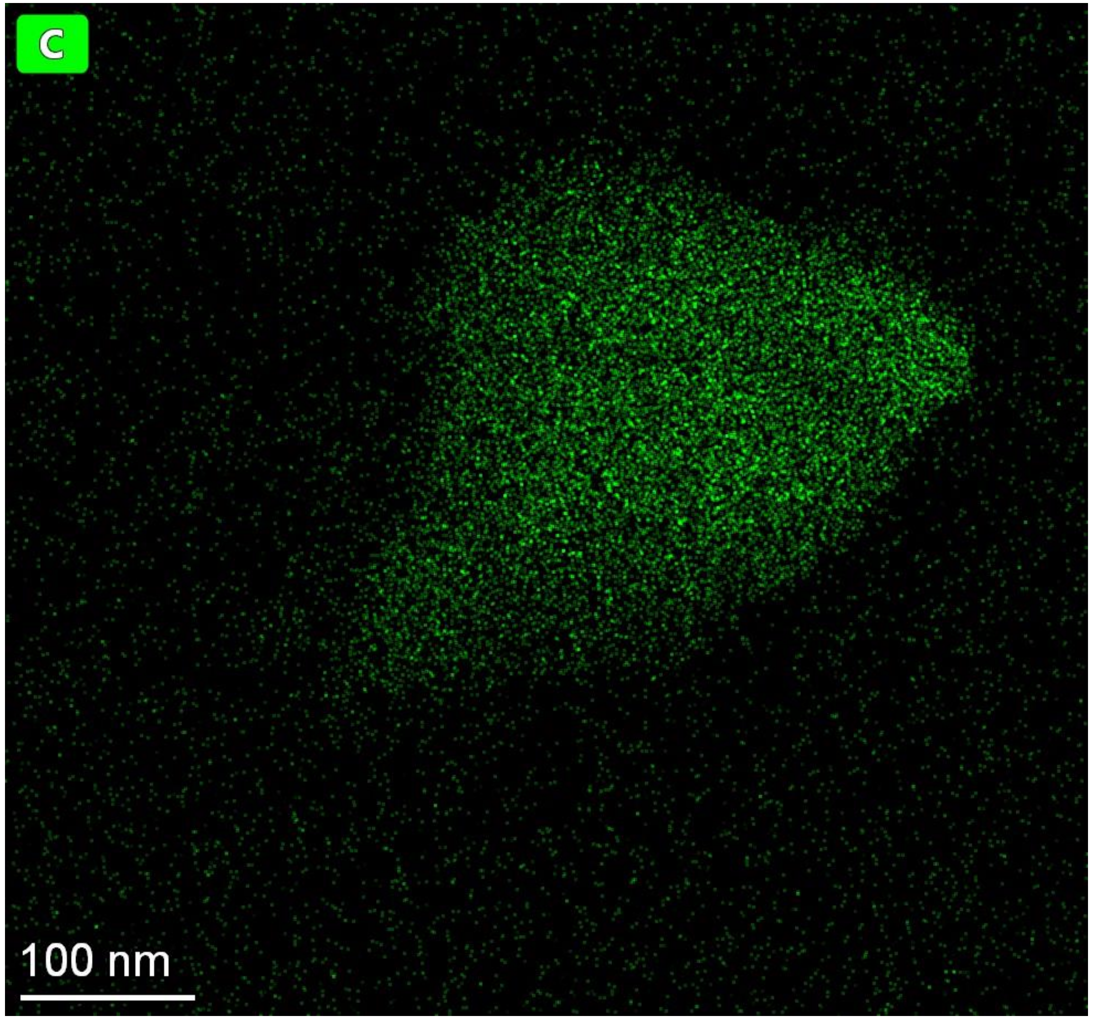

Supplementary Information-3: XRD Patterns of Bare BAC

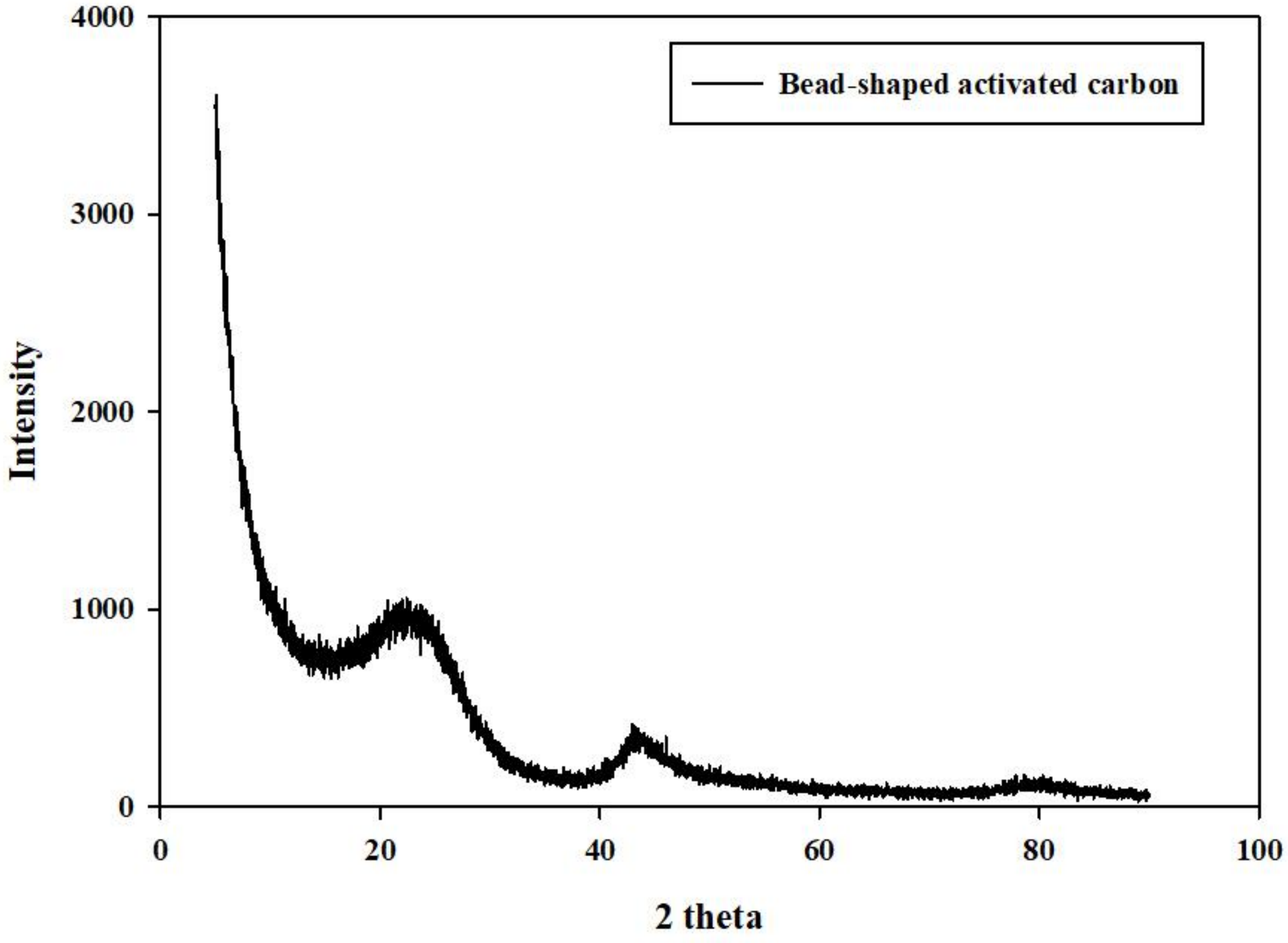

Supplementary Information-4: SEM-EDX of Bare BAC

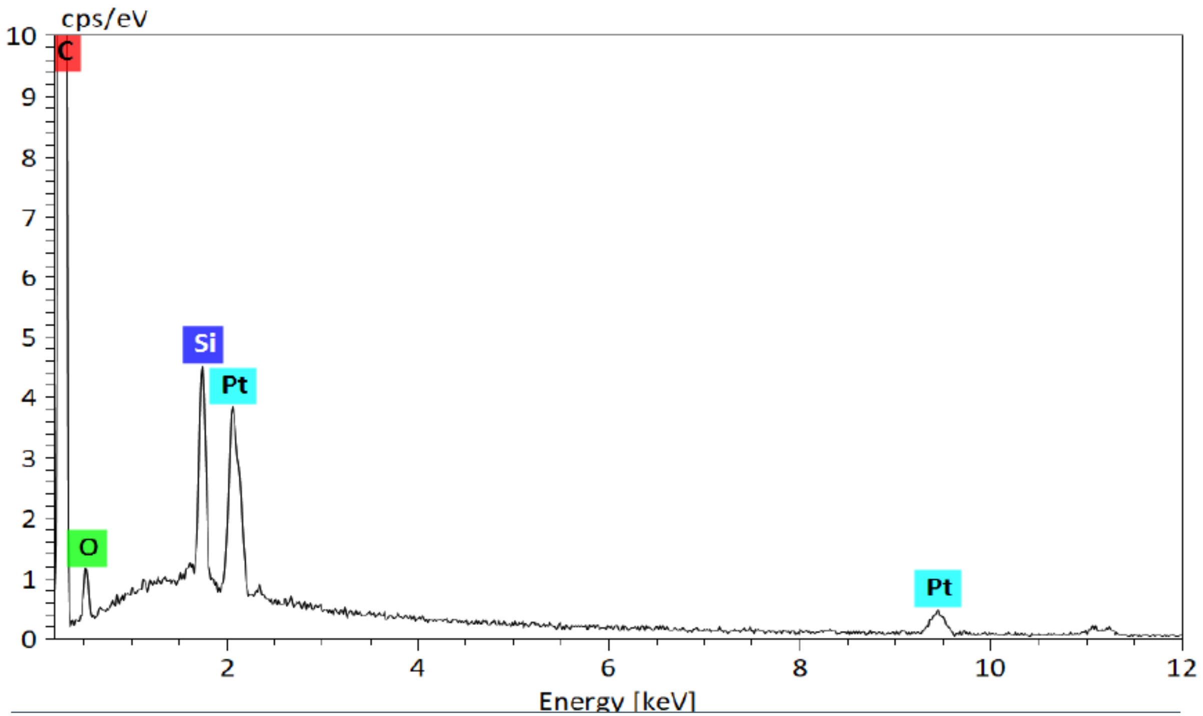

Element	Mass (%)	Atom (%)
C	94.41	96.10
O	4.45	3.40
Si	1.14	0.50
Pt	0.00	0.00
	100.00	10.00

Pt: Coating material during the measurement

Supplementary Information-5: Attrition and Abrasion Test of Bare BAC (Physical Stability)

[%] / sample	1st-time	2nd-time	Mean Value±Deviations
BAC	99.65	99.88	99.77±0.11

- Method : ASTM D4058-96 & SPENCE method, Attrition and abrasion testers
- Conditions
  Wight of test BAC : 100g
  Duration of the test : 30min
  Rotating speed : 54rpm
  Sieve : 250um

Supplementary Information-6: Acetaldehyde Cycle Test over Bare BAC

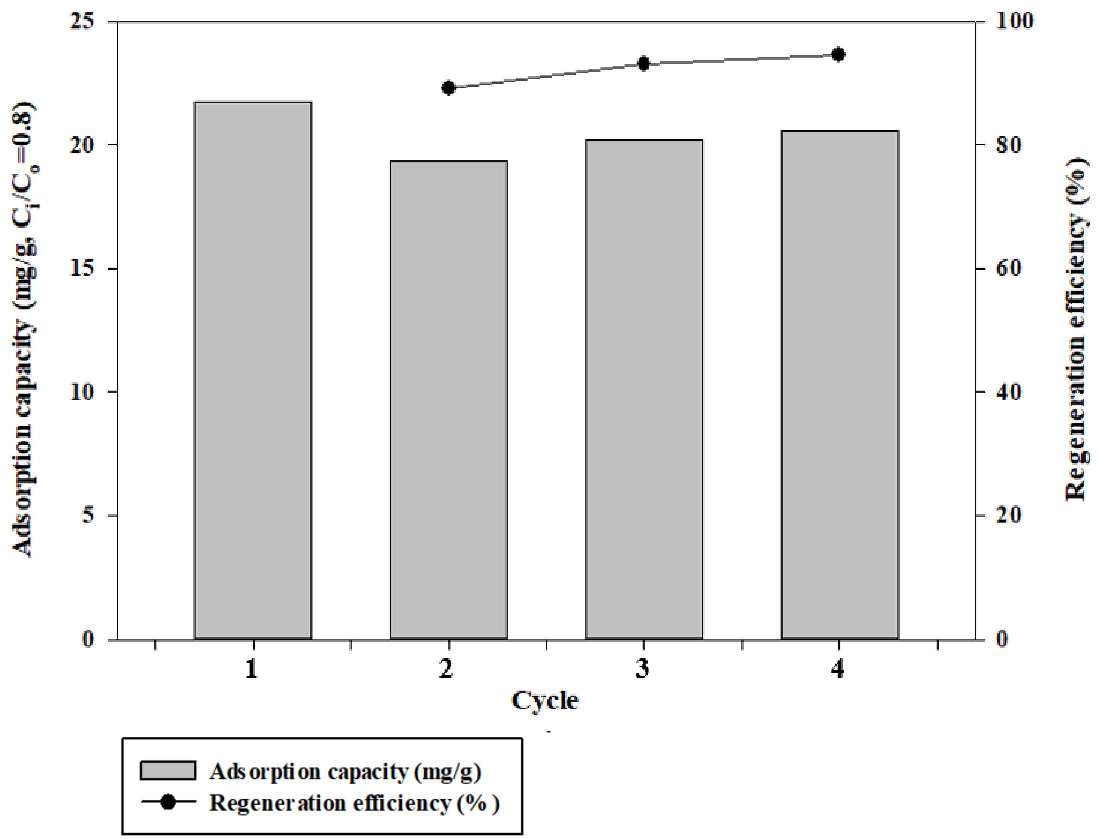

Cycle	Adsorption capacity (mg/g, C_i/C_o=0.8)	Regeneration efficiency (%)
1 (Fresh)	21.73	-
2	19.36	89.09
3	20.21	93.01
4	20.55	94.57

Acetaldehyde 200 ppm, GHSV 37,500 hr^{-1}, 25°C, 1.255 L/min, adsorbent 2 ml

Supplementary Information-7: Particle Size Distribution of Bare BAC

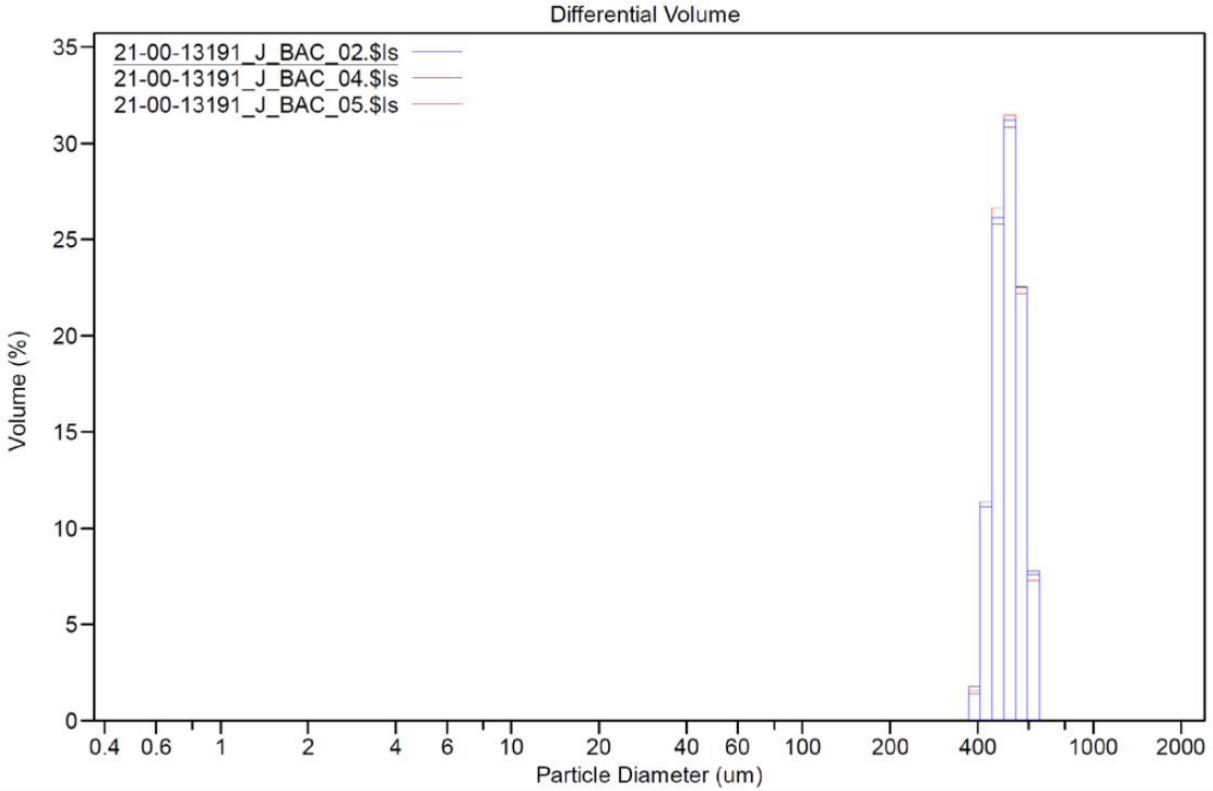

Run	Particle size (㎛)			
	Diameter at 10%	Diameter at 50%	Diameter at 90%	Average
1	440.2	511.0	589.2	512.9
2	438.7	511.0	589.6	512.7
3	440.6	510.3	588.3	512.3
SD	1.00	0.40	0.67	0.31
CV (%)	0.23	0.08	0.11	0.006
Average	439.8	510.8	589.0	512.6

Supplementary Information-8: CH_3CHO adsorption parameters on coconut shell and coal based activated carbon samples

Sample	C_{in}	W_{ad}
	[ppm]	[mg/g]
Coconut shell activated carbon	200	20.87
Coconut-P7N1-900	200	53.46
Coal based activated carbon	200	16.81
Coal-P7N1-900	200	23.43
